# Psychological Stress and Hospitalization for Childhood Asthma-a Nationwide Cohort Study in Two Nordic Countries

**DOI:** 10.1371/journal.pone.0078816

**Published:** 2013-10-25

**Authors:** Xiaoqin Liu, Jørn Olsen, Esben Agerbo, Wei Yuan, Sven Cnattingius, Mika Gissler, Jiong Li

**Affiliations:** 1 Section for Epidemiology, Department of Public Health, Aarhus University, Aarhus, Denmark; 2 Department of Epidemiology and Social Science on Reproductive Health, Shanghai Institute of Planned Parenthood Research, WHO Collaborating Center for Research in Human Reproduction, National Population & Family Planning Key Laboratory of Contraceptive Drugs and Devices, Shanghai, China; 3 Department of Epidemiology, Fielding School of Public Health, University of California, Los Angeles, Los Angeles, California, United States of America; 4 National Centre for Register-Based Research, Aarhus University, Aarhus, Denmark; 5 CIRRAU-Centre for Integrated Register-based Research, Aarhus University, Aarhus, Denmark; 6 Clinical Epidemiology Unit, Department of Medicin Solna, Karolinska University Hospital, Karolinska Institute, Stockholm, Sweden; 7 THL (Terveyden ja hyvinvoinnin laitos) National Institute for Health and Welfare, Information Department, Helsinki, Finland; 8 NHV (Nordisk högskola för folkhälsovetenskap) Nordic School of Public Health, Gothenburg, Sweden; Leiden University Medical Center, Netherlands

## Abstract

**Objective:**

Exposures to psychological stress in early life may contribute to the development or exacerbation of asthma. We undertook a cohort study based on data from several population-based registers in Denmark and Sweden to examine whether bereavement in childhood led to increased asthma hospitalization.

**Methods:**

All singleton children born in Denmark during 1977-2008 and in Sweden during 1973-2006 were included in the study (N=5,202,576). The children were followed from birth to the date of first asthma hospitalization, emigration, death, their 18^th^ birthday, or the end of study (31 December 2007 in Sweden and 31 December 2008 in Denmark), whichever came first. All the children were assigned to the non-bereaved group until they lost a close relative (mother, father or a sibling), from when they were included in the bereaved group. We evaluated the hazard ratio (HR) of first hospitalization for asthma in bereaved children using Cox proportional hazards regression models, compared to those who were in the non-bereaved group. We also did a sub-analysis on the association between bereavement and first asthma medication.

**Results:**

A total of 147,829 children were hospitalized for asthma. The overall adjusted HR of asthma hospitalization in bereaved children was 1.10 (95% confidence interval (CI): 1.04-1.16), compared to non-bereaved children. The risk of asthma hospitalization was increased in those who lost a close relative at age of 14-17 years (HR=1.54, 95% CI: 1.23-1.92), but not in younger age groups. The association between bereavement and asthma hospitalization did not change over time since bereavement. In the sub-analysis in singleton live births during 1996-2008 recorded in the DMBR, bereavement was associated with a lower use of asthma medication (HR=0.87, 95% CI: 0.80-0.95).

**Conclusions:**

Our data suggests that psychological stress following bereavement in late adolescence is associated with an increased risk of asthma hospitalization or lowers the threshold for asthma hospitalization.

## Introduction

Asthma is among the most common chronic diseases in children in high income countries with substantial cost for the health system [1,2], and one of the leading causes of school absenteeism and pediatric hospitalization [3,4]. In the Nordic countries, around 0.2% children less than 15 years old were hospitalized at least once due to asthma in 1999 [2]. 

Asthma often has an early onset [5], indicating that early-life experiences may influence the risk of asthma, but its etiology is only partly known. Psychological stress in childhood has been proposed to cause or exacerbate asthma [6,7], by having a direct effect on the Hypothalamic-pituitary-adrenal (HPA) axis and modulating the release of the steroid cortisol [8], which may increase IgE level and cause a shift in the T_H_1/T_H_2 balance toward a T_H_2-dominant immunity [9,10]. Or psychological stress may contribute to unfavorable parenting practices [11], e.g. influencing the asthma management [12], which may worsen asthma symptom. Stress exposures may also change social and environmental exposures that play a role in the etiology of asthma.

Several studies indicate an association between psychological stress and childhood asthma [13–21], but most of these studies are weak. As children’s asthma may lead to psychological stress among caregivers [22], cross-sectional or case-control studies may be subject to recall bias and reverse causation [20,21,23]. Studies with short-term or incomplete follow-up may not be able to detect whether psychological stress triggers asthma symptom or causes asthma [13–19]. Moreover, the measurement of stress is often based on the perception of stress which is modified by individual coping capacity [24]. 

Diagnosis of asthma, especially mild asthma, in children is difficult because the asthma symptoms in young children are often non-specific [25]. Wheezing symptoms in children under three years of age are common and many get prescribed anti-asthmatic medications [26]. These wheezing symptoms are often transient and including the transient wheezes in the asthma measures may dilute possible associations [27]. We used asthma hospitalization as the diagnostic indicator in our study which may reflect severe asthma cases and therefore be more specific.

Death of a close relative (mother, father or sibling) is considered to be one of the most stressful life events [28]. We hypothesized that death of a close relative in childhood increased the risk of hospitalization for asthma and a short induction time would indicate a triggering effect, and a long induction time would suggest a causal effect. We evaluated such hypotheses in a large study following children from birth until 18 years of age.

## Methods

### Ethics statements

The study was approved by Danish Data Protection Agency and Scientific Ethics Committee of Central Region Jutland in Denmark and Research Ethics Committee (EPN) at Karolinska Institute in Sweden. No informed consent is needed for register-based study according to the legislation in Denmark and Sweden.

### Study population

We conducted a cohort study using data from several population-based registers in Denmark and Sweden [29]. All live births and new residents in the two countries are assigned a unique personal identification number, which can be used to link information at the individual level in all national registers [29–32]. We first identified all singleton live births during 1977-2008 in the Danish Medical Birth Registry (DMBR) and during 1973-2006 in the Swedish Medical Birth Register (SMBR) [33]. The DMBR has been computerized since 1973 and includes data on gestational age from 1978. The DMBR was linked to the Danish Civil Registration System [30], the Danish National Hospital Register [34], the Danish Registers of Causes of Death [35], The Danish Integrated Database for Longitudinal Labor Market Research [36], and The Danish National Prescription Registry [37]. The SMBR was established in 1973. The SMBR was linked to the Swedish Multi-generation Register [38], the Swedish Cause of Death Register [39], the Swedish Patient Register [40], and the Swedish Education Register [41]. A total of 5,202,576 births were included during the study period from the two birth registries; 1,924,824 of these children were born in Denmark (37.0%) and 3,277,752 in Sweden (63.0%).

### Exposure

Exposure was defined as the loss of a mother, a father or a sibling before the children reached 18 years of age. We treated exposure as a time-dependent variable: all the children were assigned to the non-bereaved cohort at birth until they lost a close relative. From that point in time, they were moved to the bereaved cohort. We obtained information on bereavement from the Danish Registers of Causes of Death and the Swedish Cause of Death Register. Causes of death were coded according to the Danish and Swedish versions of the International Classification of Diseases (ICD). During the study period, the eighth (ICD-8) and tenth (ICD-10) revisions were used in Denmark, and ICD-8, ICD-9 and ICD-10 revisions were used in Sweden. In the bereaved cohort, 4.0% of bereaved children experienced more than one loss during the follow up. When multiple losses happened, the first bereavement was used to classify the exposure status of the children. 

### Outcome

The outcome was first hospitalization for asthma as recorded in the Danish National Hospital Register and the Swedish Patient Register. The Danish National Patient Registry contains prospectively collected information on all hospitalizations in the country since 1977; outpatient diagnoses are included from 1995 onwards [34]. The Swedish Patient Register has collected information on inpatient care since 1964/1965 and covers all hospitalizations and diagnoses since 1987 [42]. Asthma was identified based on the following ICD codes: 493(ICD-8; ICD-9); and J45, J46 (ICD-10). The first hospitalization was defined as the date of first admission in the registers for those who had one of the mentioned ICD codes.

### Follow-up

The children were followed from birth to the first of the following events: first hospitalization for asthma, emigration, death, their 18^th^ birthday, or the end of follow up (31 December 2007 in Sweden and 31 December 2008 in Denmark). 

### Covariates

The following potential confounders were included in our analysis [43–46]: sex of the child (boy, girl), country of residence (Denmark, Sweden), low birth weight (<2500g, ≥2500g), calendar year of birth (1973-1977, 1978-1982, 1983-1987, 1988-1992, 1993-1997, 1998-2002, 2003-2008), maternal age at delivery (15-26, 27-30, 31-59 years), maternal parity (1^st^, 2^nd^ and higher), mother’s country of origin (Nordic countries, other countries), maternal social status at birth (not in labor market, unskilled workers, skilled workers and white collar workers, top level status), and family history of asthma (in father, mother or sibling). Information on sex of the child, calendar year of birth, country of residence, birth weight, maternal parity, and maternal age at delivery was obtained from the DMBR and the SMBR. Information on maternal social status and mother’s country of origin was obtained from The Danish Integrated Database for Longitudinal Labor Market Research and the Swedish Education Register. We obtained information on family history of asthma from the Danish National Hospital Register and the Swedish Patient Register if they had been hospitalized after the start of the registers. 

### Statistical analysis

The data were analyzed in STATA (version 11.2), using Cox proportional hazards regression model to estimate the hazard ratio (HR) with a 95% confidence interval (CI), to assess the association between bereavement during childhood and the risk of hospitalization for asthma. To examine whether the associations between childhood bereavement and hospitalization for asthma depended on the type of loss, we categorized the bereaved children into three groups (loss of a mother, father or a sibling). We also categorized the bereaved children by age at exposure (0-3 years, 4-6 years, 7-13 years, and 14-17 years). The follow-up time was divided into four periods (≤1 year, 2-5 years, 6-10 years, and >10 years). Missing values were included in the models as a separate group.

We further categorized the cause of death into two groups: a) unexpected death (Danish codes: ICD-8: 795, E800-E999; ICD-10: R95-97, V00-Y99; Swedish codes: ICD-8: 7959, 79621, E807-E999; ICD-9: 798, E807-E999; ICD-10: R95, R96, R98, V01-Y98), and b) other causes of death. For other causes of death, psychological stress may start long before the death of a relative, thus the potential causal effect may start before the death of a relative. We carried out a sub-analysis by sub-grouping causes of death. As the association between bereavement and hospitalization for asthma may differ among ethnicities, we repeated our analysis restricted to children born to mothers of Nordic origin. To exclude the effect of multiple losses during the exposure period, we repeated our analysis restricted to children who only experienced one loss. In order to assess the impact of bereavement on asthma treatment, we also did sub-analysis of the association between bereavement and asthma medication in singleton live births during 1996-2008 recorded in the DMBR. Information on asthma medication was obtained from The Danish National Prescription Registry. The anatomical therapeutical chemical (ATC) codes for inhaled asthma drugs were: inhaled β_2_-agonists (R03AC02, R03AC03, R03AC04, R03AC12 and R03AC13), inhaled glucocorticoids (R03BA01, R03BA02 and R03BA05), fixed-dose combination of inhaled β_2_-agonists and glucocorticoids (R03AK06 and R03AK07) and leukotriene receptor antagonists (R03DC03). 

## Results

Table 1 shows the demographic characteristics of children in the two groups. In the bereaved group, there were more children born with low birth weights, who had a family history of asthma, and who were born in earlier calendar years. Mothers of bereaved children tended to be older, had a higher parity (2^nd^ and higher) and poorer social status. The two groups did not differ substantially with respect to country of residence, mother’s country of origin or sex of the child.

**Table 1:** Characteristics of the study population.

Characteristic	Bereaved		Non-bereaved	
	N	%	N	%
Country of residence				
Denmark	61,868	36.7	1,862,956	37.0
Sweden	106,781	63.3	3,170,971	63.0
Mother’s country of origin				
Nordic countries	158,905	94.2	4,708,963	93.5
Other countries	9,073	5.4	310,092	6.2
Unknown	671	0.4	14,872	0.3
Maternal age at delivery				
15-26	63,211	37.5	1,863,982	37.0
27-30	42,448	25.2	1,498,551	29.8
31-59	62,976	37.3	1,671,105	33.2
Unknown	14	0	289	0
Maternal parity				
1	63,304	37.5	2,194,969	43.6
≥2	105,157	62.4	2,837,224	56.4
Unknown	188	0.1	1,734	0
Maternal social status				
Not in labor market	28,619	17.0	748,489	14.9
Unskilled workers	35,113	20.8	990,532	19.7
Skilled workers and white collar workers	35,395	21.0	1,322,567	26.3
Top level status	19,511	11.6	717,839	14.2
Unknown	50,011	29.6	1,254,500	24.9
Sex of the child				
Boy	86,149	51.1	2,585,028	51.3
Girl	82,500	48.9	2,448,899	48.7
Low birth weight(<2500g)				
Yes	8,552	5.1	167,559	3.3
No	152,475	90.4	4,733,260	94.0
Unknown	7,622	4.5	133,108	2.7
Calendar year				
1973-1977	29,049	17.2	533,873	10.6
1978-1982	38,585	22.9	697,910	13.9
1983-1987	36,389	21.6	697,733	13.9
1988-1992	35,709	21.2	844,914	16.8
1993-1997	19,221	11.4	802,344	15.9
1998-2002	7,792	4.6	729,746	14.5
2003-2008	1,904	1.1	727,407	14.4
Family history of asthma				
Yes	18,608	11.0	443,653	8.8
No	150,041	89.0	4,590,274	91.2
Children with asthma				
Yes	1,455	0.9	146,374	2.9
No	167,194	99.1	4,887,553	97.1

The mean follow-up time was 12.9 years, and the total follow-up time was 6.7×10^7^ person-years. We observed 147,829 children who were hospitalized for asthma during follow-up, of which 92,572 were under 3 years of age, 21,962 at 4-6 years, 26,029 at 7-13 years, and 7,266 at 14-17 years. Of these, 1,455 children had been bereaved and 146,374 had not. The adjusted HR of hospitalization for asthma was 1.10 (95% CI: 1.04-1.16) in the bereaved group, compared with the non-bereaved group. The HRs were 1.05 (95% CI: 0.99-1.12) for Denmark and 1.19 (95% CI: 1.08-1.33) for Sweden. The loss of a close relative significantly increased the risk of hospitalization for asthma in children who lost a close relative at age of 14-17 years (HR=1.54, 95% CI: 1.23-1.92), but not in children who lost a close relative at other ages (table 2).

**Table 2:** Hazard ratios (HRs) for asthma hospitalization in childhood according to bereavement.

Bereavement	Cases	Person- year	Crude HRs	Adjusted HRs*(95%CI)
Non-bereaved	146,374	6.6×10^7^	1	1.00(ref)
All loss	1,455	1.2×10^6^	1.12	1.10(1.04-1.16)
Age at the time of exposure				
0-3 years of age	641	4.1×10^5^	1.05	1.03(0.95-1.11)
4-6 years of age	329	2.8×10^5^	1.14	1.08(0.97-1.21)
7-13 years of age	399	4.2×10^5^	1.16	1.09(0.99-1.21)
14-17 years of age	86	7.7×10^4^	1.62	1.54(1.23-1.92)

* Adjusted for country, mother’s country of origin, maternal age at delivery, maternal parity, maternal social status at birth, low birth weight, sex of the child, calendar year of birth, and family history of asthma

The association between bereavement and asthma hospitalization did not change over follow up time since bereavement. The HRs for asthma hospitalization for children who lost a close relative at ages of 14-17 years were 1.64 (95% CI: 1.20-2.25) in the first year following bereavement and 1.48 (95% CI: 1.08-2.03) during two to five years following bereavement (table 3).

**Table 3:** Hazard ratios (HRs) for asthma hospitalization according to bereavement stratified on age at the time of exposure and latency time since bereavement.

Age at the time of exposure	Cases	Person- year	Crude HRs	Adjusted HRs*(95%CI)
0-3years of age				
Non-bereaved	146,374	6.6×10^7^	1	1 (ref)
Latency time since bereavement				
≤1 year	109	3.2×10^4^	0.87	0.89(0.74-1.08)
2-5 years	256	1.2×10^5^	1.06	1.08(0.96-1.23)
6-10 years	165	1.3×10^5^	1.09	1.05(0.89-1.22)
>10 years	111	1.3×10^5^	1.20	1.16(0.96-1.41)
4-6 years of age				
Non-bereaved	53,996	4.7×10^7^	1	1 (ref)
Latency time since bereavement				
≤1 year	46	2.7×10^4^	1.08	1.06(0.79-1.43)
2-5 years	144	1.0×10^5^	1.15	1.17(0.99-1.38)
6-10 years	97	1.1×10^5^	1.08	1.00(0.82-1.24)
>10 years	42	4.5×10^4^	1.35	1.27(0.92-1.75)
7-13years of age				
Non-bereaved	32,264	3.4×10^7^	1	1 (ref)
Latency time since bereavement				
≤1 year	78	6.6×10^4^	1.20	1.20(0.96-1.51)
2-5 years	221	2.3×10^5^	1.15	1.11(0.97-1.28)
6-10 years	99	1.2×10^5^	1.17	1.07(0.87-1.33)
>10 years	1	2.8×10^3^	0.53	0.60(0.08-4.28)
14-17years of age				
Non-bereaved	6,871	1.0×10^7^	1	1 (ref)
Latency time since bereavement				
≤1 year	45	3.6×10^4^	1.83	1.64(1.20-2.25)
2-5 years	41	4.1×10^4^	1.45	1.48(1.08-2.03)

* Adjusted for country, mother’s country of origin, maternal age at delivery, maternal parity, maternal social status at birth, sex of the child, low birth weight, calendar year of birth, and family history of asthma

We estimated HRs of hospitalization for asthma according to bereavement stratified on age at the time of exposure and categories of deceased relatives. Similar associations according to categories of deceased relatives were observed in children who lost a close relative at ages of 14-17 years (Table 4). 

**Table 4:** Hazard ratios (HRs) for asthma hospitalization according to bereavement stratified on age at the time of exposure and categories of deceased relatives.

Age at the time of exposure	Cases		Person- year	Crude HRs	Adjusted HRs*(95%CI)
0-3years of age					
Non-bereaved		146,374	6.6×10^7^	1	1 (ref)
Categories of deceased relatives					
Death of mother		83	6.3×10^4^	0.86	0.82(0.66-1.02)
Death of father		301	1.8×10^5^	1.13	1.12(1.00-1.26)
Death of a sibling		257	1.7×10^5^	1.03	1.06(0.93-1.20)
4-6 years of age					
Non-bereaved		53,996	4.7×10^7^	1	1 (ref)
Categories of deceased relatives					
Death of mother		56	5.3×10^4^	1.03	0.96(0.73-1.26)
Death of father		163	1.3×10^5^	1.21	1.15(0.98-1.35)
Death of a sibling		110	9.6×10^4^	1.12	1.14(0.94-1.38)
7-13years of age					
Non-bereaved		32,264	3.4×10^7^	1	1 (ref)
Categories of deceased relatives					
Death of mother		69	1.0×10^5^	0.85	0.78(0.91-1.00)
Death of father		245	2.3×10^5^	1.34	1.25(1.10-1.43)
Death of a sibling		85	9.5×10^4^	1.09	1.15(0.92-1.43)
14-17years of age					
Non-bereaved		6,871	1.0×10^7^	1	1 (ref)
Categories of deceased relatives					
Death of mother		20	2.0×10^4^	1.43	1.25(0.78-2.02)
Death of father		54	4.4×10^4^	1.78	1.75(1.33-2.31)
Death of a sibling		12	1.3×10^4^	1.38	1.34(0.72-2.50)

* Adjusted for country, mother’s country of origin, maternal age at delivery, maternal parity, maternal social status at birth, sex of the child, low birth weight, calendar year of birth, and family history of asthma

There was no statistically significant difference in HRs between children who lost a close relative unexpectedly and who lost a close relative due to other causes of death (data not shown). We got similar results when we restricted analysis in children born to mothers of Nordic origin (data not shown). The HR was 1.13 (95% CI: 1.06-1.19) when we restricted our analysis to children who experienced only one loss. 

In the sub-analysis in singleton live births during 1996-2008 recorded in the DMBR, we found that the adjusted HR for asthma hospitalization was 0.92 (95% CI: 0.78-1.08) and asthma medication 0.87 (95% CI: 0.80-0.95) in the bereaved group, compared with the non-bereaved group. 

## Discussion

In this population-based study, we observed that bereavement in childhood was associated with a modest increased risk of hospitalization for asthma, which was mainly contributed by the increased risk in children who lost a close relative at age of 14-17 years. Bereavement was associated with a lower use of asthma medication.

The magnitude of association we observed in adolescent was similar to Lietzen’s finding in adult population, which found individuals whose family member had severe illness experienced 1.5-fold risk of asthma compared with individuals without stressful life events [47]. Our findings support the biopsychosocial model of stress, which suggests that psychological stress may have an effect on the immune system, altering cytokine production in the direction of Th-2 response [9], and thus may evoke asthma exacerbation [48]. We found the bereaved children were less likely to be prescribed asthma medication in childhood, thereby, it is possible that the increased risk of asthma hospitalization is a result of insufficient treatment [12]. It is also possible that the threshold for hospitalization is changed by the bereavement. Depressed caregivers may feel unable to cope with childhood asthma and ask for hospitalization.

Children’s understanding of death and their coping mechanisms are closely related to the age-dependent developmental capacity [49–52]. Children under three years of age probably do not understand the concepts of death [53]. Children at an advanced age may perceive bereavement more stressful than children at a very young age, thereby, exhibit greater inflammatory signals in the lung and increased severity of asthma [54]. In addition, asthma by different ages at onset may be of different phenotypes [55,56]. It is therefore also possible that our finding may reflect the association between bereavement and different phenotypes of asthma. Asthma that had a late onset is often non-atopic and more severe [57], and is more sensitive to psychological stress [58].

Compared with previous studies, our study has a number of strengths. Our study was population-based cohort with almost complete long-time follow-up. The exposure data was collected independent of the occurrence of outcome, making differential misclassification unlikely. Additionally, information on deaths in the registers is of high quality and loss of a close relative is an objective and well-defined source of stress. The risk of exposure misclassification is therefore small. Furthermore, the large sample size in our study presented a unique opportunity to detect even small differences between the groups.

Our study also has limitations. When multiple losses happened during the follow-up period, we used the first bereavement to define the age at exposure. We repeated our analysis by excluding those who had experienced more than one loss, and the results were similar. As we used hospital discharge diagnosis as the outcome of interest, only severe asthma was included in our study. It is likely that some of those first admissions at older ages in the register were among children who already had asthma at earlier ages but not being hospitalized. Thereby, it is possible that some of the effects shown by us are associated with lower thresholds to hospitalization due to stress exposure. Parental smoking is likely to be causally related to asthma in childhood [59,60], and we had no data on that. If smoking causes premature death and bereaved children are expected to be more exposed to smoke, it would bias our results towards positive findings. The association we have reported, therefore, needs to be interpreted with caution. Another limitation is that we did not adjust for infections in our multivariate analysis as there are evidences indicating a relation between psychological stress, infections, and asthma [14,61]. If infections in early life are in the pathway between bereavement and asthma, they should not be adjusted for [62]. 

## Conclusions

Our findings suggest that psychological stress in late adolescence increases the risk of asthma hospitalization or lowers the threshold for asthma hospitalization. 
